# Theory of Mind: Did Evolution Fool Us?

**DOI:** 10.1371/journal.pone.0087619

**Published:** 2014-02-05

**Authors:** Marie Devaine, Guillaume Hollard, Jean Daunizeau

**Affiliations:** 1 Motivation, Brain, and Behavior Laboratory, Brain and Spine Institute, Hôpital de la Pitié Salpêtrière Paris, France; 2 Maison des Sciences Economiques, Paris, France; 3 CNRS UMR 7225, INSERM U 975, UPMC Paris, France; 4 Wellcome Trust Centre for Neuroimaging, University College London, London, United Kingdom; Ecole Normale Supérieure, France

## Abstract

Theory of Mind (ToM) is the ability to attribute mental states (e.g., beliefs and desires) to other people in order to understand and predict their behaviour. If others are rewarded to compete or cooperate with you, then what they will do depends upon what they believe about you. This is the reason why social interaction induces recursive ToM, of the sort “I think that you think that I think, etc.”. Critically, recursion is the common notion behind the definition of sophistication of human language, strategic thinking in games, and, arguably, ToM. Although sophisticated ToM is believed to have high adaptive fitness, broad experimental evidence from behavioural economics, experimental psychology and linguistics point towards limited recursivity in representing other’s beliefs. In this work, we test whether such apparent limitation may not in fact be proven to be adaptive, i.e. optimal in an evolutionary sense. First, we propose a meta-Bayesian approach that can predict the behaviour of ToM sophistication phenotypes who engage in social interactions. Second, we measure their adaptive fitness using evolutionary game theory. Our main contribution is to show that one does not have to appeal to biological costs to explain our limited ToM sophistication. In fact, the evolutionary cost/benefit ratio of ToM sophistication is non trivial. This is partly because an informational cost prevents highly sophisticated ToM phenotypes to fully exploit less sophisticated ones (in a competitive context). In addition, cooperation surprisingly favours *lower* levels of ToM sophistication. Taken together, these quantitative corollaries of the “social Bayesian brain” hypothesis provide an evolutionary account for both the limitation of ToM sophistication in humans as well as the persistence of low ToM sophistication levels.

## Introduction

Theory of Mind (ToM) is the ability to attribute mental states (e.g., beliefs and desires) to other people in order to understand and predict their behaviour [1]. This ability lies at the core of human social cognition: it develops early in life [2], and its impairment is associated with severe neuropsychiatric disorders [3]–[4]. ToM endows us with highly adaptive social skills, such as teaching, persuading or deceiving [5]. Thus, natural selection should have promoted phenotypes that exhibit highly sophisticated forms of ToM [6]–[10].

In fact, behavioural economics has provided undisputable experimental evidence of people’s *bounded rationality* in strategic interactions [11]. In particular, we seem to be very limited in our ability to correctly guess the behaviour of others in games [12]–[14]. These results corroborate experimental psychology studies [15]–[16], as well as linguistic and even literary evidence [17]–[18] that all point towards a heterogeneous and limited ToM sophistication in humans. We may thus wonder why evolution has not made all of us smarter. In particular, what made it possible for low ToM sophistication phenotypes to persist in socially demanding environments? In this work, we test whether such apparent limitations may not in fact be proven to be adaptive, i.e. optimal in an evolutionary sense. In turn, this raises two challenging issues: (i) how do we formally define ToM sophistication phenotypes?, and (ii) how do we measure their adaptive fitness?

We start with the premise that if others are rewarded to compete or cooperate with you, what they believe you will do is relevant for you to predict their behaviour. This is the reason why social interaction induces recursive thinking, of the sort “I think that you think that I think, etc.”. Critically, recursion is the common notion behind the definition of sophistication of human language [19]–[20] and strategic thinking in games [14], [21]. In line with Yoshida et al. [22], we define ToM sophistication as the depth of recursive thinking. Here, a 0-ToM agent learns (over the course of repeated interactions) how likely her opponent’s choices are. In contrast, a 1-ToM agent adopts the “intentional stance” [23], i.e. she tries to understand how 0-ToM updates his belief, from observing his behaviour. Hence, 1-ToM is defined in terms of her recursive belief, i.e. her belief about 0-ToM’s belief. A 2-ToM observer assumes she faces either a 1-ToM or a 0-ToM agent. This means she has to both recognize the sophistication of her opponent and understand how he learns. More generally, a k-ToM agent tries to understand how her opponent learns, under the assumption that he is less sophisticated than herself. In so doing, k-ToM forms high-order recursive beliefs, which may be highly uncertain. Thus, we model the impact of subjective uncertainty onto the mechanism of belief update using information theory (cf. the *Bayesian brain* hypothesis [24]–[26]).

In the context of social interaction, we are left with the question of what prior information agents use to learn about how others learn. Here, we simply assume that the brain’s model of other brains presumes they are optimal too. By this we mean that people believe other conspecifics behave according to common sense (e.g., they make decisions that reveal their preferences and beliefs, which change as learning unfolds). The key idea here is to consider how such common sense notion impacts on the (Bayes-optimal) learning rules of agents interacting with each other. In this context, Bayes-optimality simply means that information processing suffers no distortion aside from potential prior biases. Agnostic priors on peoples’ choices (i.e. priors that do not involve the intentional stance) would yield Bayesian agents that track the descriptive statistics of others’ choices. This is essentially what 0-ToM learners do. Eventually, they arrive at uncertain estimates (beliefs) of, e.g., others’ choice frequency. However, Bayes-optimal forecasts of 0-ToM’s behaviour rely on the (ambiguous) identification of the covert beliefs and preferences that determine her overt decisions. This is the essence of 1-ToM’s learning rule, which relies on an informative prior assumption, namely: others are (agnostic) Bayes-optimal agents. Under this “social Bayesian brain” hypothesis, one can derive the learning rule of k-ToM agents recursively, starting with 0-ToM (see **Models**).

Although k-ToM learners are all Bayes-optimal, they differ in terms of the depth of recursion of their beliefs. This difference in ToM sophistication changes the way k-ToM agents react to a given sequence of their opponent’s action. For example, 0-ToM will tend to act as if her opponent was more likely to pick the action that she had chosen most frequently in the past. In turn, 1-ToM will anticipate this and act accordingly. Since their respective behavioural response pattern will be different, 2-ToM is in a position to discriminate between 0-ToM and 1-ToM (and act accordingly). In brief, k-ToM will best-respond to her opponent’s past choices, under the constraint of limited sophistication. Thus, ToM sophistication phenotypes are characterized in terms of (formal) belief update rules that (i) are specific to the depth of their recursion, and (ii) shape their behavioural strategy over the course of repeated social interactions.

We address the second challenge from the perspective of evolutionary game theory (EGT). In brief, EGT states that the reproductive and survival successes of any behavioural phenotype is determined by how well it performs when interacting with other alternative phenotypes [27]. Here, we extend this idea to evaluate the adaptive fitness of ToM sophistication. Current ethological debates highlight the importance of competitive versus cooperative types of reciprocal social interactions in the evolution of ToM [10]. We thus focused on a pair of two-players games that capture these two canonical forms of social interaction. In “hide and seek”, the gain of the winner is exactly balanced by the loss of the looser, which is the essence of competition. In contradistinction, agents playing “battle of the sexes” are most rewarded for coordinating their behaviour (see **Models and Methods**). Note that both games’ payoffs are contingent on players’ ability in predicting their opponent’s behaviour (there is no prior good decision).

## Results

### On the Relative Performance of ToM Phenotypes Engaged in Iterated Games

**Figure 1:**
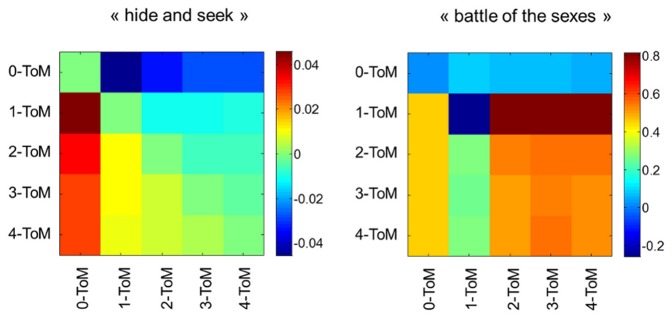
MCMC average payoffs of all pairs of ToM agents. This figure depicts the MCMC average of the payoff matrices for both “hide and seek” (left) and “battle of the sexes” (right) after learning has occurred. The i^th^ line gives the accumulated payoff of the i^th^ type of agent, when playing against each and every other ToM phenotype. Note that the absolute payoff levels of both types of games cannot be compared.

To assess the relative performance of ToM sophistication phenotypes engaged in either cooperative or competitive social interactions, we performed the following series of Monte-Carlo simulations. We let all 5×5 = 25 combinations of pairs of ToM agents (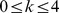) play repeatedly “hide and seek” and “battle of the sexes” (cf. game outcomes in Table 1 below) against each other. One simulation thus consisted of the history of beliefs, choices and outcomes, for both agents, across trials (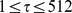).We measured the accumulated payoff each ToM phenotype receives as a function of trial τ, when interacting with any other ToM phenotype. We repeated each type of simulation 500 times, in order to average out variability arising from behavioural noise (see Methods section below). Figure 1 depicts these payoff matrices at trial τ = 512. Since τ controls the amount of available information, those can be understood in terms of the relative success of ToM phenotypes after learning has occurred.

**Table 1:** Payoffs for each player in the “hide and seek” game (left) and “battle of the sexes” (right).

	P2: 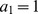	P2: 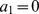		P2: 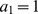	P2: 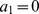
P1: 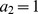	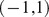	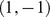	P1: 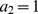		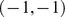
P1: 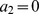	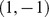	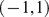	P1: 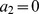	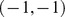	

Numbers inside brackets indicate the payoffs; the number on the left (resp. on the right) indicates the payoff player 1 (resp. player 2) gets when making decision 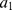 while player 2 chooses 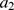.

In the competitive game, the expected payoff matrix is anti-symmetrical (this is because “hide and seek” is a zero-sum game). Overall, increasing ToM sophistication improves performance: for any ToM level, gains are systematically positive (respectively, negative) against less (respectively, more) sophisticated ToM agents. Interestingly, there is a systematic cost to sophistication: the relative gains decrease as the difference in ToM levels increases. This informational cost to sophistication essentially limits the way one can exploit less sophisticated ToM agents. Results in the context of the cooperative game are entirely different. Here, pairs of agents with different ToM levels perform much better than pairs of “twin” 0-ToM and 1-ToM agents, who fail to coordinate their behaviour. Note that the best performance level is observed for 1-ToM agents, when playing against *more* sophisticated agents. In addition, behavioural performance of pairs of k-ToM agents with k≥2 neither depends upon whether agents have similar sophistication levels (“twin” pairs versus non “twin” pairs), nor on the sophistication level *per se*. This is surprising, since it suggests that there is no advantage in being more sophisticated than a 2-ToM agent when engaging in a cooperative interaction. This means that being less sophisticated than the other player is only detrimental (in the sense of yielding inaccurate behavioural predictions) in a competitive setting.

**Figure 2:**
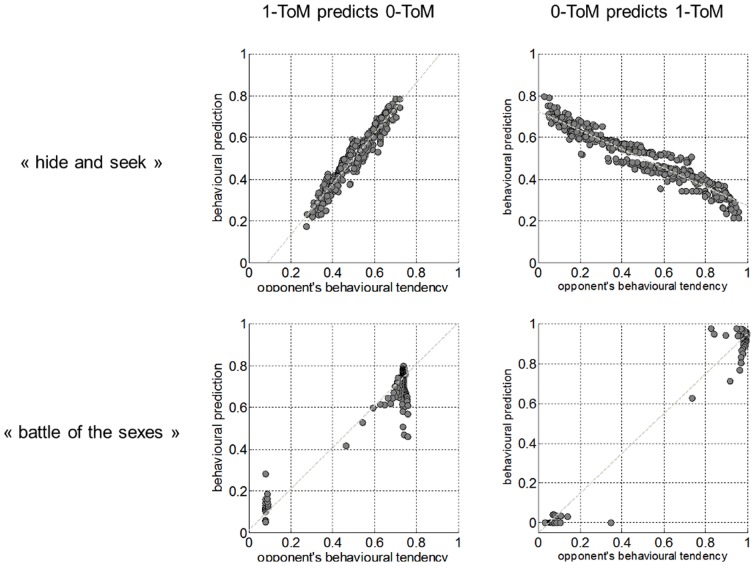
Accuracy of behavioural predictions in competitive and cooperative contexts: example of 0-ToM playing against 1-ToM. The behavioural prediction 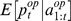 of ToM players (y-axis) is plotted against her opponent’s true behavioural tendency 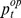 (x-axis) for each trial of a simulated repeated game with 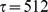 trials. The grey line indicates the best-fitting straight line in the data. Upper half: “Hide and Seek”. Lower half: “Battle of the Sexes”. Left: accuracy of 1-ToM predictions when playing against 0-ToM. Right: accuracy of 0-ToM predictions when playing against 1-ToM.

The nature of the beliefs, which ToM agents develop as learning unfolds during the iterated games, sheds some light on these intriguing results.Recall that 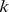-ToM selects the appropriate action 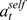 on the basis of her prediction 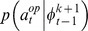
about her opponent’s next move. Figure 2 compares this prediction against the real behavioural tendency experienced by her opponent, in the case of 0-ToM playing against 1-ToM (for both games).

One can see that when playing “hide and seek”, 1-ToM predicts very well the behaviour of 0-ToM, but that 0-ToM is almost always entirely wrong about 1-ToM next move. In other words, 0-ToM agents are fooled by 1-ToM agents in a competitive setting.

**Figure 3:**
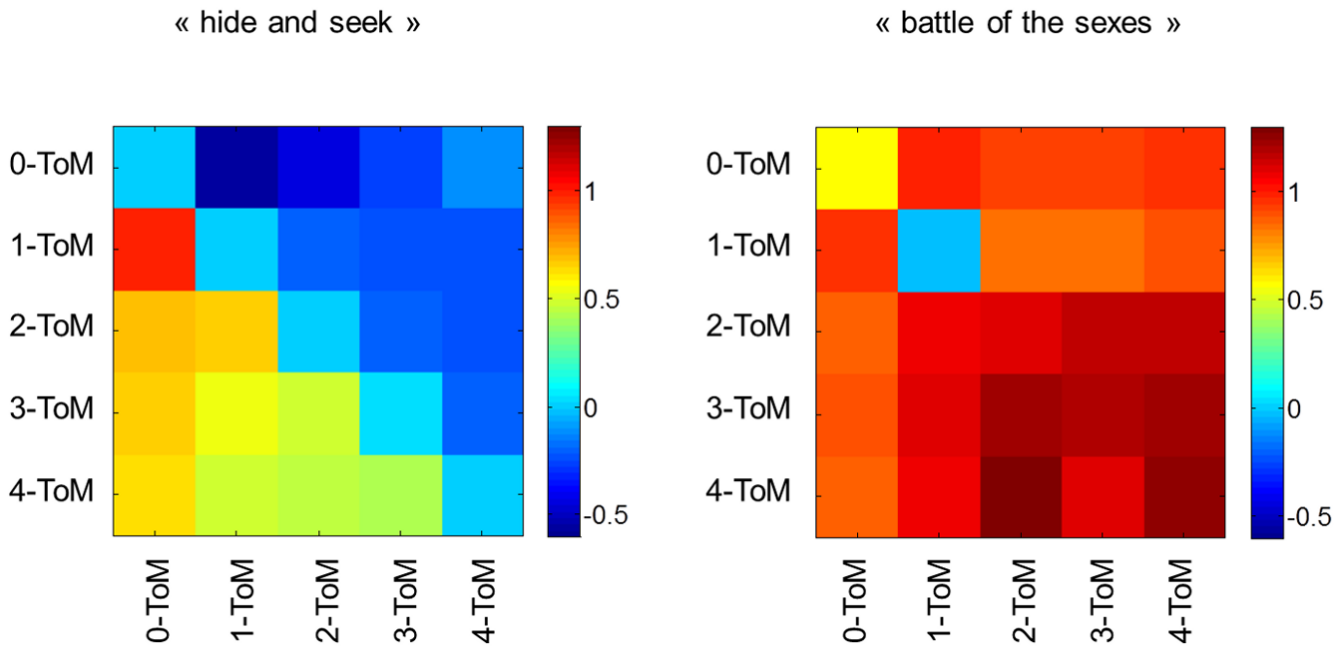
MCMC average prediction accuracy of all pairs of ToM agents. This figure depicts the MCMC average of the linear trend between the behavioural prediction 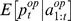 of ToM players and their opponent’s true behavioural tendency 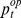. In other words, this corresponds to the slope of the best-fitting straight line in Figure 2. The figure uses the same format as Figure 1.

However, this is not the case when ToM agents play “battle of the sexes”: both players are able to correctly predict the behaviour of their partner. In other words, 0-ToM is not confused by 1-ToM in a cooperative setting. We will now check whether this difference between the prediction accuracy of less sophisticated ToM agents in a competitive/cooperative context generalizes to any ToM sophistication level. Figure 3 summarizes the quality of this behavioural prediction for all pairs of ToM players.

One can see that the overall pattern is quite similar to the behavioural performances depicted on Fig. 1. This is intuitive, since this means that the accuracy of the prediction determines a significant amount of the variability in behavioural performance.

This is particularly salient when ToM agents play “hide and “seek”, which induces an almost perfect anti-symmetric pattern in the prediction accuracy. This means that, on average, ToM agents are fooled by more sophisticated opponent in a competitive setting. Note that “twin” pairs (pairs of ToM agents with identical sophistication levels) form behavioural predictions that are, on average, uncorrelated with the real behavioural tendency of their opponent. In addition, the prediction accuracy decreases with the ToM sophistication level. This consequence of statistical complexity induces the cost to sophistication that was observed on behavioural performance or accumulated reward (cf. Fig. 1).

These results are somewhat at odds with the pattern of prediction accuracy of ToM agents playing “battle of the sexes”. In brief, except for “twin” pairs of 0-ToM and 1-ToM agents, behavioural predictions are quite accurate. Interestingly also, behavioural predictions slightly improve with overall ToM sophistication level. This means that, on average, ToM agents are not confused by more sophisticated partners in a cooperative setting. In fact, ToM agents even benefit from the sophistication of their partner. This holds as well for “twin” pairs of 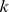-ToM agents, provided 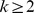. This is important, since this means that being less sophisticated than the other player is only inappropriate (in the sense of yielding inaccurate behavioural predictions) in a competitive setting.

**Figure 4:**
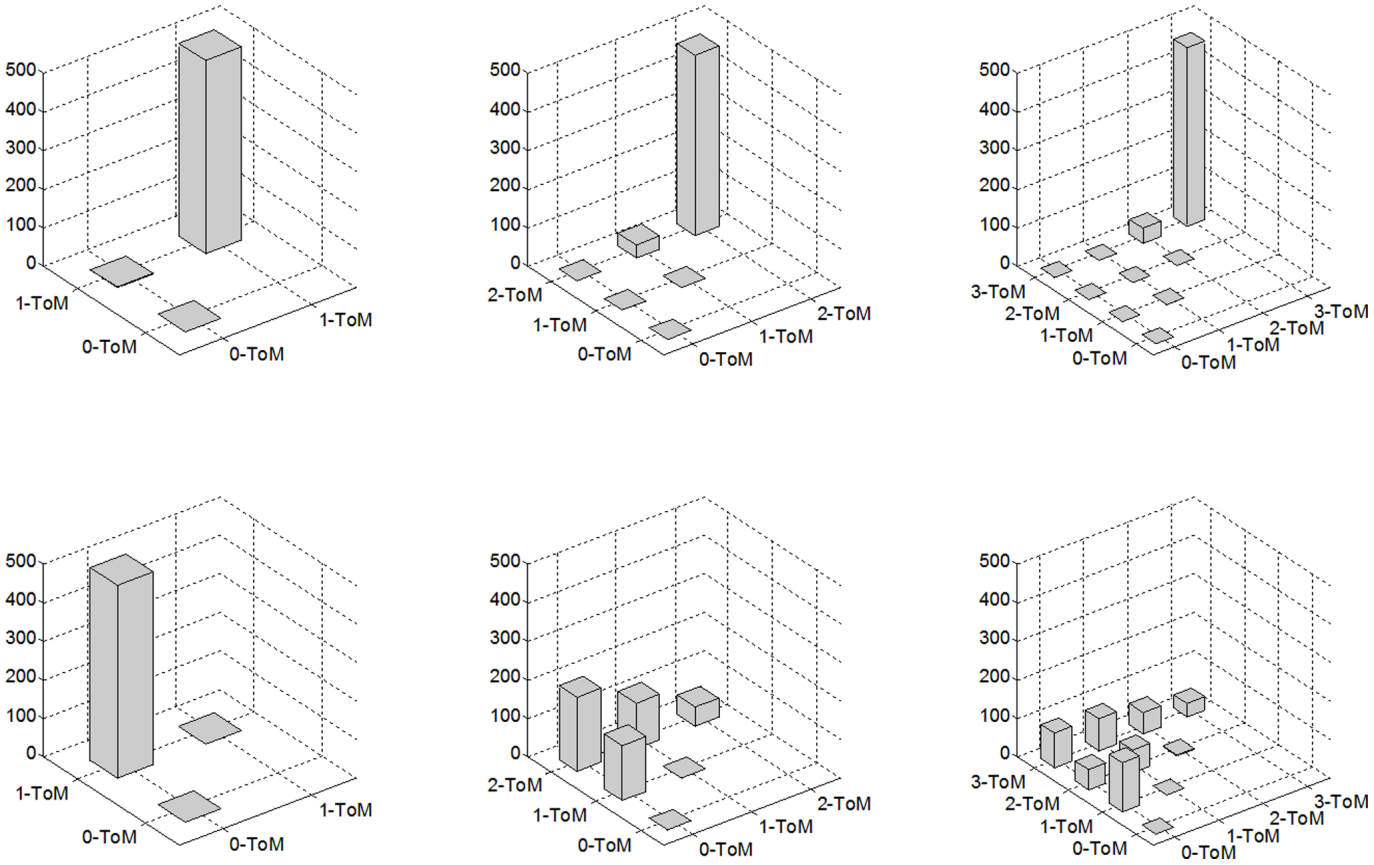
MCMC empirical distribution of learned opponent’s sophistication level for “twin” pairs of ToM agents. Each bar gives the number of MCMC simulations (z-axis) that led to each particular combination of belief 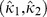, both agents had on each other’s ToM sophistication level (x/y-plane). Histograms are truncated to the upper-left triangle for visualization purposes (they are symmetrical by construction). Upper half: “Hide and Seek”. Lower half: “battle of the Sexes”. Left: “twin” pairs of 2-ToM agents, Middle: “twin” pairs of 3-ToM agents, right: “twin” pairs of 4-ToM agents.

The case of “twin” pairs is interesting because it reveals a fundamental difference between the nature of beliefs in competitive and cooperative contexts. In brief, for 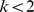, “twin” pairs form poor behavioural predictions about their opponent, whether they are in a competitive or in a cooperative context. More precisely, their behavioural predictions are effectively non-informative (they are right half of the time). However, for 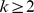, 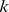-ToM agents that engage in a cooperative context can form very accurate behavioural predictions. Recall that 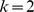 is a critical ToM sophistication level, in that any 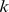-ToM agent with 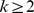 has to learn the sophistication level of the other player. It turns out this is quite important to understand the difference in the prediction accuracy of “twin” pairs of 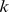-ToM agents (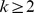) in a competitive or a cooperative context, respectively. We will now summarize the beliefs of “twin pairs” of 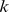-ToM agents about their opponent’s sophistication, and highlight its impact on behavioural performance in both games. First note that 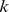-ToM agents in a “twin pair” cannot infer the correct level of their opponent. This is because, by construction, they assume their opponent is less sophisticated than themselves. However, we will see that the type of game is highly predictive of the nature of their (erroneous) inference. Figure 4 depicts the MCMC empirical histograms of ToM sophistication levels (see **Models and Methods**) attributed by “twin” pairs of 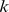-ToM agents with 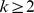 to each other, for both types of games.

One can see that when playing “hide and seek”, each 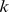-ToM agent in the “twin” pairs almost always believes that her opponent is a 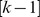-ToM agent (cf. peak at the upper-right corner of the histograms). In other words, the competitive setting induces a bias in the attribution of the opponent’s ToM level towards maximal sophistication.

Results are entirely different when ToM agents play “battle of sexes”. In this context, a pair of “twin” 2-ToM agents eventually arrives at different beliefs: one agent believes her opponent is 0-ToM, whereas the other systematically thinks hers is 1-ToM (cf. peak at the upper-left corner of the histogram). This makes the “twin” 2-ToM behave as a pair of 1-ToM and 2-ToM agents, and yields good coordination performance (cf. Fig. 1). This pattern tends to be confirmed for “twin” pairs of 3-ToM and 4-ToM: the agents almost never have the same belief about their opponent sophistication (cf. empty main diagonal in the histograms). In fact, agents have heterogeneous beliefs most of the time, which makes them behave as a heterogeneous pair. In other words, the cooperative setting induces a bias towards heterogeneous reciprocal beliefs about each other ToM sophistication. This means that coordination is successful when there is heterogeneity in the reciprocal beliefs about ToM sophistication levels. Ironically speaking, successful cooperation arises when one agent is more dismissive about her partner than her partner is about her.

To sum up, in contrast to competitive interactions, ToM agents are not confused by more sophisticated partners in a cooperative setting. In fact, ToM agents even benefit from the sophistication of their partner.

### Evolution of ToM: Influence of Learning and Cooperative Interactions

**Figure 5:**
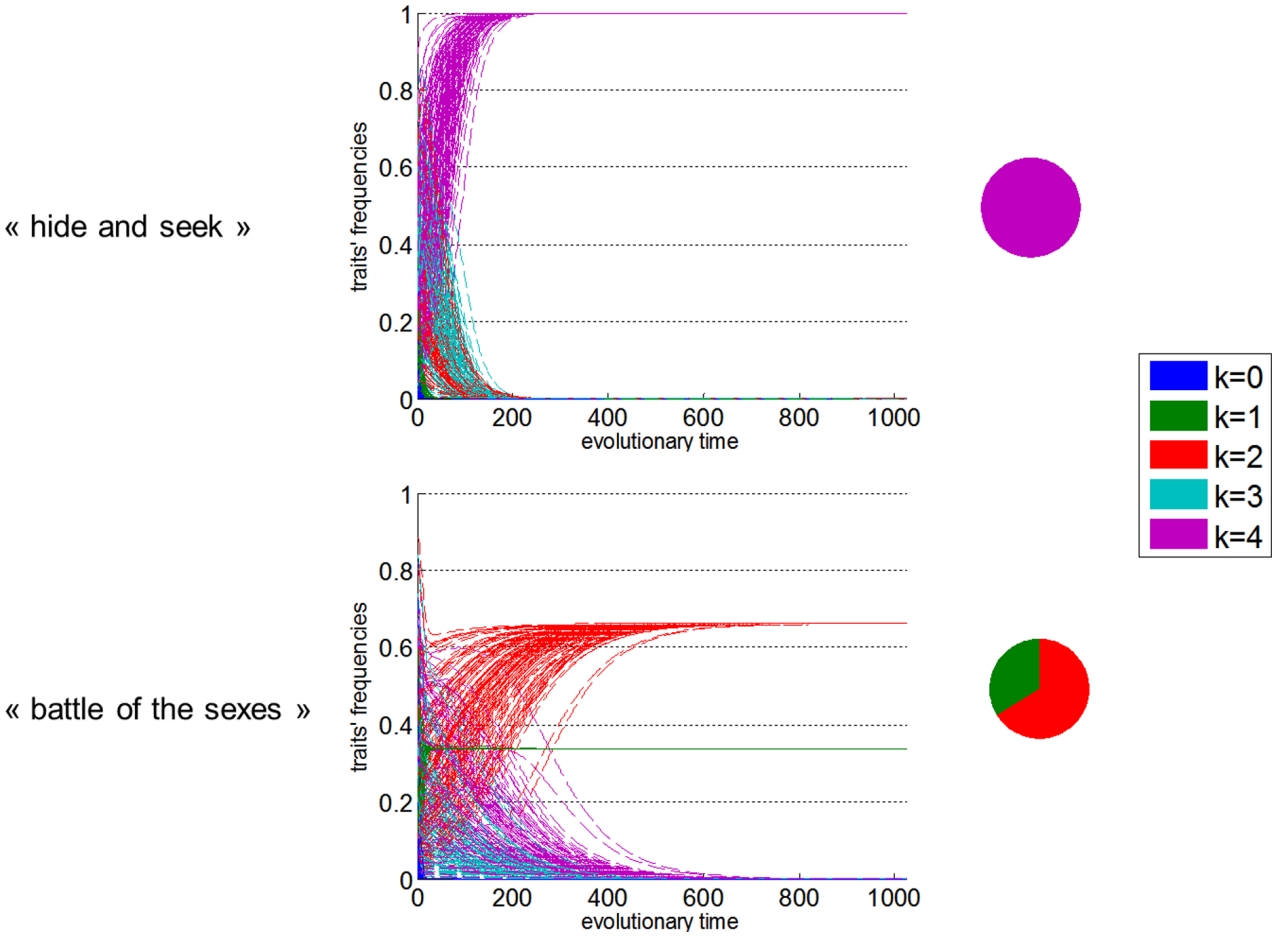
Replicator dynamics for purely cooperative and competitive social interactions. The frequency of each ToM phenotype (y-axis) is plotted against evolutionary time (x-axis), for 128 different simulations with different initial conditions. Different ToM traits correspond to different colours (see legend). Pie charts depict the evolutionary stale states, i.e. the equilibrium or fixed point, replicator dynamics converge to (the colour coding is the same). Upper half: “Hide and Seek”. Lower half: “battle of the Sexes”.

We then used EGT to simulate the evolution of societies populated with heterogeneous ToM sophistication phenotypes. In brief, we inserted the average payoff matrices into EGT replicator dynamics, which describe the dynamics of the frequency of competing phenotypes over evolutionary time (see **Models and Methods**). These eventually converge to the evolutionary stable states, which are a repartition of phenotypes that is restored by selection after a disturbance [27]. Figure 5 shows examples of replicator dynamics, with five ToM phenotypes (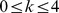), after 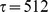 game iterations, and for both pure cooperative (“battle of the sexes”) and pure competitive (“hide and seek”) social interactions.

Different paths correspond to different initial phenotypes frequency distributions. First, one can see that the equilibrium points are stable, with basins of attraction spanning all sampled initial conditions (this was always the case). Second, these evolutionary stable states depend upon the game type (i.e. cooperative or competitive). This is because replicator dynamics unfold from relative performance of ToM phenotypes captured by payoff matrices depicted on Figure 1. In a purely competitive context, evolutionary dynamics follow a very reproducible sequence of ToM phenotypes extinction. In brief, expected extinction time increases with ToM sophistication levels, i.e. 0-ToM traits disappear first, then 1-ToM, 2-ToM, etc… This winner-take-all Darwinian competition eventually selects the most sophisticated ToM phenotype, whose evolutionary stable frequency reaches unity.

As one would expect from behavioural performance results (cf. Figure 1), replicator dynamics in the context of purely cooperative interactions are qualitatively different. In brief, two time scales seem to be at play: first, very quick selection pressure make 0-ToM disappear and the frequency of 1-ToM phenotypes converge towards 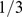. Second, slower winner-take-all competition between higher ToM sophistication phenotypes (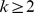) eventually selects 2-ToM phenotypes, whose evolutionary stable frequency approaches 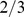.

**Figure 6:**
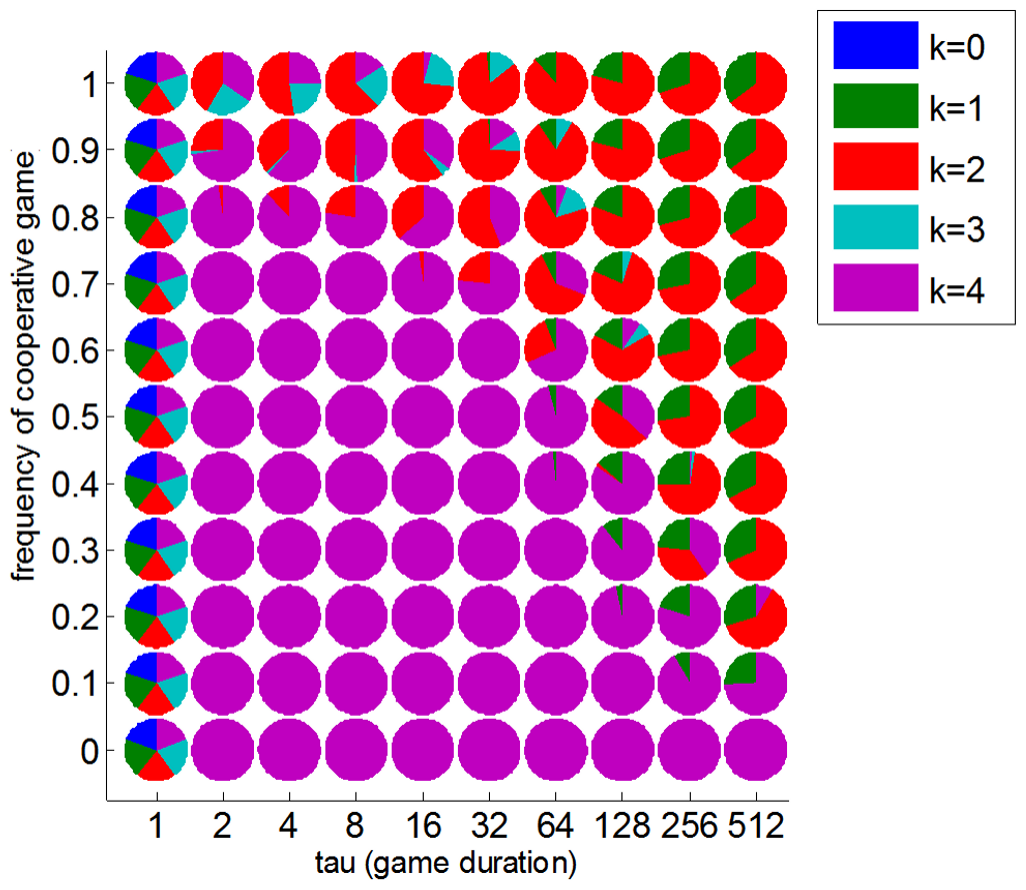
Phase diagram of ToM evolution. Each pie chart depict the evolutionary stable state that is induced by a particular combination of amount of learning τ (x-axis) and proportion ω of cooperative interactions (y-axis).

Let us now inspect in a more systematic manner the effect of cooperation and learning onto evolutionary stable states. In brief, we varied the proportion 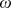 of cooperative social interactions as well as the number of game iterations 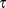 (see **Models and Methods**). Note that no oscillation or cycle in the evolutionary dynamics was observed throughout the entire range of phase parameters 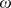 and 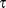. This means that selective pressure always eventually converges toward an evolutionary stable state. Additionally, this evolutionary stable state was always unique (no multistability). Taken together, this means evolutionary stable states are a faithful summary of replicator dynamics. Figure 6 summarizes the dependency of evolutionary stable states w.r.t. 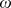 and 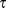.

First, irrespective of the proportion 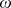 of cooperative interactions and the number of game iterations 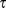 (except for one-shot games, i.e.: 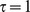), the 0-ToM phenotype is not evolutionary stable. This means that selective pressure favours phenotypes that are capable of taking an “intentional stance”. In other words, natural selection induces a lower bound on ToM phenotypes. Second, evolutionary stable states are either dominated by the most sophisticated ToM phenotypes (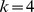) or consist of mixed populations, most particularly when cooperative social interactions become more likely. More precisely, when the proportion ω of cooperative social interactions reaches a critical threshold, the population mostly consists of ToM phenotypes smaller than 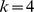. This means that cooperative social interactions effectively induce an upper bound on ToM sophistication. Note that the critical threshold depends upon the amount 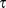 of learning: the longer the games, the smaller the proportion of cooperative social interactions is required for inducing the upper bound on ToM sophistication. Essentially however, with enough learning experience, cooperation would in most cases yield the same evolutionary stable state, namely a mixture of 1-ToM and 2-ToM phenotypes. Effectively, one can thus think of 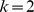 as the most likely upper bound on ToM sophistication.

**Figure 7:**
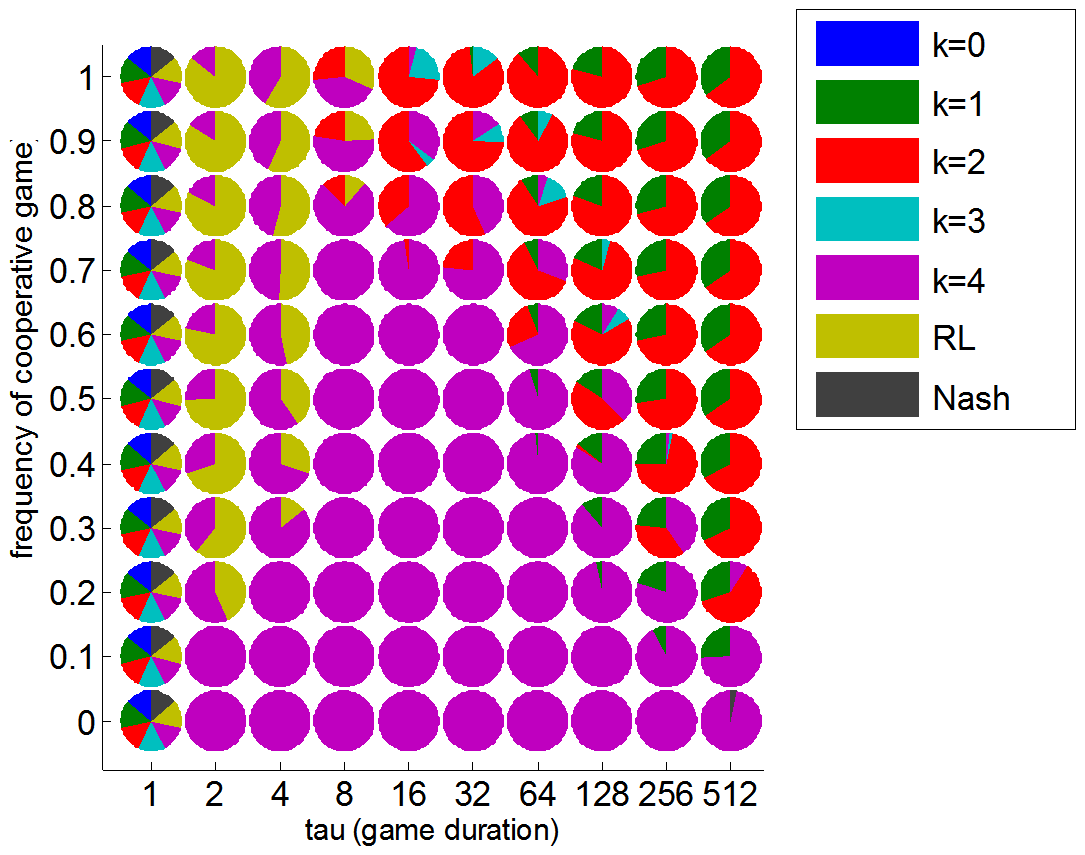
Phase diagram of ToM evolution: Impact of RL and Nash phenotypes. This figure uses the same format as Fig. 6.

One may wonder whether our main conclusion still holds if other types of players invade the population. In fact, it may be argued that behavioural responses in the context competitive or cooperative games may be driven by mechanisms that are qualitatively different from ToM. We have thus augmented the pool of possible phenotypes within our population of agents with objectively optimal strategies (i.e. Nash players) and adaptive heuristic behavioural traits (i.e. reinforcement learners). The former phenotype is motivated from game theoretic considerations: playing Nash is typically understood as the average best response (across all types of opponent’s strategies [28]. The latter phenotype is derived from behaviouristic accounts of decision making: in brief, animal act on the basis of learned action-outcome contingencies. Reinforcement learning (RL) is a celebrated model of such automatic behavioural processes [29]. Note that RL generalizes “tit-for-tat” or “win-stay, loose-switch” heuristic strategies, which have been suggested to be of particular importance for explaining the emergence of altruism and cooperation in evolving human societies [30]. Figure 7 depicts the ensuing replicator dynamics phase diagram, having included Nash and RL agents within the set of competing phenotypes.

One can see that including Nash and RL agents does not fundamentally change the overall picture. Interestingly, there is no combination of cooperation and learning that make the Nash phenotype evolutionary stable. This is because, even though no other phenotype performs better than Nash on average, ToM phenotypes achieve higher performance when facing each other. Second, only in the context of very short games can the RL phenotype be considered evolutionary stable: RL agents effectively disappear for game durations longer than 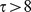. This is actually the only noticeable difference with Figure 6: short game durations, which were previously dominated by the highest ToM sophistication level (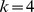), now yield mixed populations that include RL phenotypes. However, the critical threshold on the amount of cooperation (above which less sophisticated ToM phenotypes dominate) is unchanged. In addition, the nature of evolutionary stable states above this critical threshold seems to be invariant to the presence or absence of non-ToM phenotypes. This includes the induced lower and upper bounds on ToM sophistication levels.

## Discussion

In this work, we have proposed a quantitative evolutionary account of ToM sophistication in humans. This relies upon a meta-Bayesian formalism [26] for recursive ToM inferences that arise in the context of reciprocal social interactions. The key idea here is that meta-bayesian agents learn or recognize the subjective (potentially high-order) beliefs of other agents in a Bayes-optimal fashion. Here, ToM sophistication is defined as the level of recursion of such meta-bayesian agents. We have assessed the relative performance of ToM agents playing competitive or coordinative games with each other. Finally, we have identified what evolutionary forces could have led to the observed variability of ToM sophistication in humans. More precisely, we have shown that: (i) a non-trivial informational cost to sophistication limits the way one can exploit less sophisticated ToM agents, and (ii) one may benefit from engaging in a cooperative interaction with more sophisticated ToM agents. Eventually, these properties yield an evolutionary stable mixture of ToM phenotypes with a lower bound at k = 1 (agents without ToM get extinct) and an upper bound at k = 2.

Our model was largely inspired by previous work from behavioural economics and experimental psychology on bounded rationality. More precisely, *k*-ToM shares with models such as “level k” [13] and the “cognitive hierarchy” [12] the notion of recursive thinking. These models have been typically used to explain people’s behaviour in non-repeated games such as the “beauty contest” (but see [14], [31]–[32] for nice extensions to repeated games). They prove useful in capturing inter-individual variability in peoples’ behaviour, in terms of the sophistication of their strategic thinking. For example, Camerer and colleagues [21] have reported the following distribution of levels: around 20% of level 0 players, 33% of level 1, 25% of level 2 and then a decreasing proportion of higher levels. Although not identical, such results are consistent with our EGT prediction (cf. the distribution peaks around level 1 and 2). Observed discrepancies may have three distinct causes. First, peoples’ behaviour is not unambiguously mapped onto levels of strategic thinking (cf. issues with levels’ stability across games, etc…). Second, we may not have included all the relevant evolutionary constraints on ToM sophistication (see comment below on comparing ToM across species). Third, there are conceptual differences between *k*-ToM (which deals with the sophistication of learning rules) and the cognitive hierarchy (which cares about the sophistication of behavioural policies). This theoretical difference is not trivial. On the one hand, one could argue that the basic cognitive resource that underlies both processes is the same, namely: the ability to form recursive beliefs. On the other hand, theory of mind is essentially inferential (cf. the intentional stance). That is, ToM is engaged when we identify mental states (beliefs, intentions, emotions, etc…) from social signals (decisions, facial expressions, etc…). In this perspective, ToM may have more to do with the way we adapt to others (through learning) than with the evaluation of the consequences of our actions (decision making).

We will now discuss the limitations of our model.

First, we did not account for social preferences or norms, such as fairness or inequity aversion. These are thought to explain people’s altruistic behaviour despite strong incentive for betrayal, as in the “prisoner’s dilemma” game [33]–[34]. However, it turns out that, in these games, meta-Bayesian agents choose the egoistic (dominant) strategy, irrespective of their ToM sophistication level. This means that ToM alone cannot explain people’s altruistic behaviour. Interestingly, a recent study [35] has used EGT with the iterated “prisoner’s dilemma” to explain the emergence of fairness through evolution. The captivating question of whether ToM’s adaptive fitness depends upon social preferences (and reciprocally) is beyond the scope of the present work. Addressing this would require modelling, e.g. inference on others’ fairness preferences.

Second, our approach shares with similar hierarchical models (such as the “cognitive hierarchy” [13], [21]) the relative arbitrariness of the first level. This is critical, because the behavioural response of all subsequent levels in the hierarchy (recursively) rely on the definition of the first level [36]. Our definition of 0-ToM agents follows from the “Bayesian brain” hypothesis: there is no reason to consider 0-ToM agents that would not learn optimally, aside from their inability to take the “intentional stance”. We believe this is mandatory for evaluating ToM’s adaptive fitness. This is because we do not want the effect of ToM sophistication on behavioural performance to be confounded by differences in, e.g., the principles underlying the way agents learn and decide. Taken together, these considerations constrain the definition of 0-ToM agents. This deserves further comments. It seems to us that it would not make sense to define 0-ToM agents that would be insensitive to feedback (e.g., payoff). This is because there will always exist a broad class of social interactions, in the context of which any such feed-forward system would perform very poorly. In other terms, feed-forward 0-ToM agents would have no evolutionary adaptive fitness. Critically, the feedback’s source is twofold: context (i.e. nature of the interaction -cf. game payoff table-) and opponent (i.e. behavioural tendencies). This is important, because there are not many types of agents that would differ qualitatively in their response to such information. An example of an agent sensitive to the context but not to her opponent is the Nash policy. By construction however, the ensuing 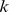-ToM agents would be Nash players as well, and thus ToM sophistication would have no adaptive fitness. In contradistinction, imitative learners are sensitive to their opponent, but not to context. However, the adaptive fitness of such agents is similar to feed-forward agents. Yet another possibility is to consider agents that would respond to an aggregate context-opponent feedback, namely: reward. This is the essence of genuine reinforcement learning (RL) agents. Note that, in terms of behavioural performance, RL agents are comparatively closer to 0-ToM than to any other agent type we have considered (including Nash players; cf. Figure 7). In fact, this was expected, since there is a linear one-to-one mapping between the value of each option and the opponent’s choice probability. Additionally, 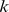-ToM agents (with 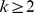) have a clear tendency to identify RL players as 0-ToM, at least in a competitive context. This means that we expect our results to be robust to re-defining 0-ToM agents as RL agents. Note that any agent that would be differentially sensitive to context and opponent feedbacks would be formally very similar to our 0-ToM. Taken together, we believe our results would be very robust to admissible changes in the definition of 0-ToM agents.

Third, one may invoke another line of work, which consists in considering that biological costs (such as brain size) induce additional evolutionary forces that eventually limited our cognitive skills [37]. The weakness of such studies is the lack of specificity: how global features such as brain size relate to different cognitive functions is unclear. In any case, what we have shown is that one does not have to appeal to biological costs to explain our limited ToM sophistication. More generally, one could challenge the very idea that natural selection acted upon ToM sophistication. For example, a radical non-adaptationist scenario would consider that such cognitive phenotypes evolved from random genetic drift. Alternatively, one could argue that ToM sophistication is a by-product of constraints imposed by other cognitive traits (such attention or working memory) that were under selective pressure. Debates about whether or not a given phenotype has been shaped by natural selection are not uncommon in evolutionary biology (cf. e.g., [38]). In our context, we would appeal to the importance of social cognitive skills in shaping humans’ adaptive fitness [7], [8]. However, we believe that, if properly extended, our work could provide a more satisfactory answer to this question. This is because EGT can be used to predict a specific relationship between features of the ecological niche (here, we considered the proportion of cooperative interactions and the typical amount of learning) and the distribution of ToM sophistication. The key point is that such features can vary across different species. Thus, provided one appropriately captures the critical differences between ecological niches, one could then test the induced variability in ToM sophistication (across species) against the null. We will pursue this in subsequent publications.

Last, one could challenge the fact that we have neglected developmental (and, to a lesser extent maybe, pathological) aspects of ToM [39]. This is related to the notion of “proximal constraints” of evolution, which relate to the ability of individuals to gradually adopt behavioural strategies that have local adaptive fitness, and are thus positively reinforced by their environment [40]. Applying the principles of such reinforcement learning theories of motivation [41] would advocate for considering agents that could change their ToM sophistication level at will. Here, we have rather assumed that ToM sophistication is a phenotype that can hardly be changed or learned over the course of the agent’s life time. However, another way of looking at ToM phenotypes is in terms of an informative prior belief on the population profile of ToM sophistication. Effectively, k-ToM phenotypes can be thought of as agents with unbounded ToM sophistication, who *a priori* believe that their conspecifics’ level of ToM sophistication cannot exceed k-1. This has two implications: (i) one could relax this prior and effectively allow agents to adapt their effective ToM sophistication level, and (ii) one could think of evolution as selecting a very specific form of prior that defines classes of meta-Bayesian agents [42].

To conclude, our meta-Bayesian approach unravelled non-trivial properties of inferential aspects of ToM. In particular, the informational cost to sophistication is a key determinant of ToM’s adaptive fitness. Note that this cost might in fact induce strong evolutionary forces for most cognitive processes that can be viewed as inferential in nature, as is the case for, e.g., learning or perception [24], [43]. This is because, as any ill-posed problem, inference heavily relies upon some form of prior information or belief [44]. Critically, we speculate that the sophistication of such prior eventually matches the complexity of the agent’s ecological niche, because of its inevitable evolutionary cost/benefit ratio.

## Models and Methods

In this section, we describe our model of theory of mind in human observers/agents. This model attempts to capture how agents infer on others beliefs and preferences, given a series of observed choices. In [26], we exposed a Bayesian solution to the inverse BDT problem (where BDT stands for “Bayesian Decision Theory”). The inverse BDT problem relates to inferring prior beliefs and subjective utility from observed decisions. This meta-bayesian approach enables us to place ToM processes on a solid quantitative footing, which obeys optimality principles. In brief, learning rules unfold from information theory. Here, we extend this approach to account for the fact that agents can differ in terms of the depth of recursivity of their beliefs (cf. “cognitive hierarchy” [21]).

### Cooperative and Competitive (Reciprocal) Social Interaction

Note that it is the reciprocal nature of social interaction that induces the potentially infinite recursion of ToM. This is because if my actions cannot influence your environment, what I believe or feel is irrelevant to you, i.e. you do not have to go beyond 0-ToM. Thus, ToM sophistication levels can only be assessed in the context of reciprocal interaction, which is why we adopt a game theoretic formulation of ToM. In its simplest form, a game is defined in terms of a utility table 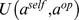, which yields the payoff one gets when making decision 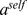 while the other player chooses 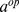. Incentives can be arbitrarily chosen to capture different forms of social exchanges or transactions, which makes game theory a very general and powerful tool to analyze the behaviour of people engaged in social interactions [28].

We aim at understanding the respective impact of cooperative and competitive (reciprocal) social interactions onto the adaptive fitness of ToM sophistication levels. We thus have to choose appropriate game-theoretic scenarios that capture these types of interaction. Critically however, we chose games whose computational challenge is similar, in the sense that payoff is contingent on how well players predict their opponent’s behaviour.

An ecologically valid proxy for a competition for resources is the game “hide and seek” (also named “matching pennies”), which has already been extensively used in experimental assessments of animal ToM, e.g. food-caching in birds [45]. In evolutionary terms, the average payoff of phenotypes playing “hide and seek” can be thought of as a proxy for survival success in the context of competitive social interactions. The version of “hide and seek” we use is a symmetric zero-sum game, whose outcome table is given in Table 1 of the main text. For any decision pair 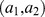, the gain of the winner is exactly balanced by the loss of the loser, which makes “hide and seek” the simplest of all conflict games. Here, the “hider” wins when 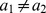 and the “seeker” wins when 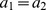. Its Nash equilibrium is a mixed strategy with probabilities 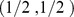 for both players. This completely random policy is the best strategy against itself, but yields an average payoff of zero. In contradistinction, bilateral deviation from Nash can induce strong bias in the expected outcomes, whereby a given strategy can be exploited by the other one.

“Battle of sexes” is a cooperation game that emulates a dilemma, whereby coordination is only achieved at the cost of one’s subjective preferences [46]. Interestingly, it is known in the animal literature as “intralocus sexual conflict”: it arises when a trait which is good for the breeding success of one sex is bad for the other [47]. More generally, the average payoff of phenotypes playing “battle of sexes” can be thought of as a proxy for mating success through (costly) cooperation. We will use a symmetric version of it, whose outcome table is also given in Table 1 of the main text. Here, players are most rewarded for coordinated behaviour (i.e., 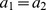), whereas they are punished when choose different options (i.e. 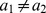). Note that, in contradistinction with “hide and seek”, payoffs are unbalanced (chance: 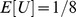). Essentially, the game payoffs are such that: (i) if one knew what the other player would do, one would choose to cooperate, and (ii) if one had no idea what the other player would do, one would choose the option that maximizes one’s own preferences. There are two pure Nash equilibria, i.e. either both players choose 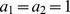, or both players choose 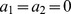. However, in both situations, one player does better than the other one (unfair outcomes). In addition, there is one Nash mixed strategy, with probabilities 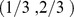 for player 1 and 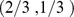 for player 2.

### Meta-Bayesian Agents

According to Bayesian decision theory, agents aim at maximising expected payoff 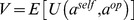, where the expectation is defined in relation to the agent’s uncertain predictions about his opponent’s next move (see below). Importantly, this implies that the form of the decision policy is the same for all agents, irrespective of their ToM level. In this work, we consider that agent’s choices may exhibit small deviations from the optimal decision rule, i.e. we assume agents employ the so-called “softmax” probabilistic policy:
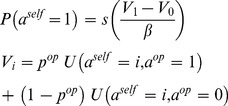
(1)where 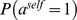 is the probability that the agent chooses the action 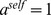, 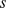 is the standard sigmoid function and 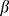 is the exploration temperature that controls the magnitude of behavioural noise. Equation 1 simply says that the probability of choosing the action 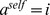 increases with its expected payoff 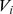. Here, the critical variable is 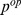: the probability that the opponent will choose the action 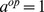.

The repeated observation of his opponent’s behaviour 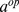 gives the agent the opportunity to learn this prediction. Theory of Mind comes into play when agents consider that the opponent’s behavioural tendency 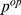 is motivated by his hidden beliefs and desires. More precisely, our “social Bayesian brain” hypothesis implies that ToM agents consider that the opponent is himself a Bayesian agent, whose decision policy 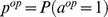 is formally similar to Equation 1. In this situation, one has to track one’s opponent’s prediction 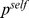 about one’s own actions. This makes ToM agents *meta*-Bayesian agents [26], i.e. Bayesian observers of Bayesian agents. In line with the notion of cognitive hierarchy [21], this meta-Bayesian inference is recursive (“I think that you think that I think…”). The recursion depth induces different ToM levels, which differ in how they update their subjective prediction 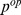.

In analogy to Yoshida et al. [22], we thus define ToM levels (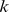-ToM agents) in terms of the way they learn from their opponent’s behaviour, starting with 0-ToM. By convention, a 0-ToM agent does not attribute mental states to his opponent. More precisely, 0-ToM agents assume that their opponents choose the action 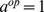 with probability 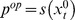, where the log-odds 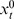 varies across trials 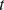 with a certain volatility 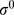 (and 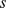 is the sigmoid function). Observing his opponent’s choices gives 0-ToM information about the hidden state 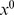, whose estimate is updated trial after trial. Under these premises, one can derive 0-ToM’s Bayesian learning rule, in terms of the change in his prediction about his opponent’s next move (see Text S1):
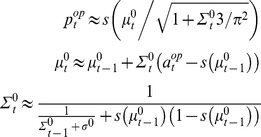
(2)where 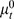 (resp. 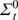) is the mean (resp. the variance) of 0-ToM’s posterior distribution 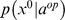 on the log-odds 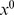, having observed his opponent’s behaviour up to trial 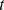. In other words, 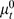 is 0-ToM’s estimate of the log-odds at trial 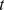, and 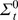 is his subjective uncertainty about it. Inserting 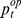 into Equation 1 now yields 0-ToM’s decision rule. Note that the term 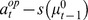 can be thought of as a prediction error, whose impact on learning accounts for changes in the subjective uncertainty 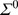. Here, the effective learning rate is controlled by the volatility 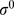, which captures 0-ToM’s priors (see [48] for a hierarchical generalization, where 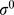 is learned as well). At the limit 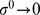, Equation 2 converges towards the opponent’s choice frequency and Equations 1–2 essentially reproduce “fictitious play” strategies [49].

Taken together, Equations 1–2 describe how 0-ToM agents learn and decide, trial by trial. This is the starting point for a 1-ToM agent, who considers that she is facing a 0-ToM agent. This means that 1-ToM has to predict 0-ToM’s next move, given his beliefs and the choices’ payoffs. The issue here is that 0-ToM’s priors (as well as his exploration temperature) are unknown to 1-ToM and have to be learned, through their non-trivial effect on 0-ToM’s choices. More precisely, 1-ToM agents assume that 0-ToM chooses the action 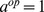 with probability 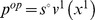, where the hidden states 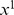 lumps 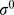 and 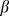 together and the mapping 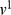 is derived from inserting Equation 2 into Equation 1. Similarly to 0-ToM agents, 1-ToM assume that the hidden states 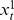 vary across trials with a certain volatility 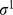, which yields a meta-Bayesian learning rule similar in form to Equation 2 (see below).

More generally, 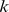-ToM agents (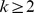) consider that their opponent is a 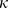-ToM agent with a lower ToM sophistication level (i.e.: 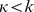). Importantly, the sophistication level 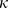 of 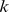-ToM’s opponent has to be learned, in addition to the hidden states 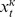 that control the opponent’s learning and decision making. The difficulty for a 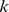-ToM agent is that she needs to consider different scenarios: each of her opponent’s possible sophistication level 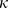 yields a specific probability 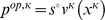 that she will choose action 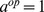.

We will now show how to derive the learning rule of a 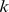-ToM (meta-Bayesian) observer, who takes the intentional stance when interpreting the behaviour of his 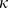-ToM opponent. Below, 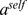 (resp. 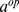) denotes 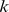-ToM’s action (resp. his 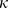-ToM opponent’s action). Let 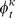 be the set of sufficient statistics that parameterize the (probabilistic) belief of a 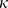-ToM observer at trial 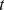 of the repeated game. Typically, the states 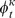 include the first- and second-order moments of the conditional probability density on the uncertain (hidden) state of his opponent (e.g., 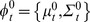 in Equation 2 above). As 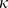-ToM learns, her belief evolves from trial to trial according to Bayes’ rule, which can be summarized as the change in the states 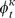 [26]:
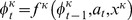
(3)where 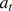 are the players’ action at trial 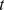 and 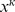 is a set of parameters that encode 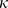-ToM’s priors. For example, the belief evolution function 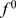 of 0-ToM is given in Equation 2. Both the derivation and the explicit form of the belief evolution function 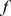 will become clearer below. At this point, suffices to say that the dynamics of belief sufficient statistics 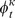 is controlled by 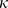-ToM’s priors 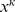. Recall that 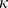-ToM’s belief serves to make a prediction 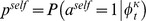 about her own move 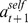 at the next trial. This then enters Equation 1 to yield 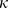-ToM’s softmax decision policy 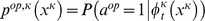, where 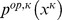 depends upon 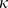-ToM’s priors 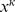 through her posterior belief’s sufficient statistics 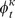. Let us first assume that 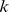-ToM knows his opponent’s sophistication level 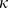, e.g. 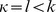. Taken together, Equations 1 and 3 then induce the following (Bernouilli) likelihood function for 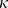-ToM’s actions sequence, *from the perspective of* 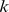 *-ToM*:
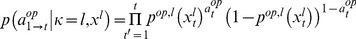
(4)where where 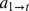 stands for all actions up to trial 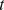, and 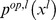 is derives from inserting Equation 3 into Equation 1. Equation 4 measures how likely is any particular history of choices up to trial 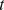, given 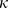-ToM’s (unknown) properties 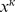, having fixed her sophistication level to 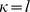. In fact, 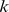-ToM does not know the level 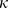 of her opponent. Without loss of generality, the complete likelihood of the actions sequence of 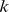-ToM’s opponent can thus be written as the following mixture:
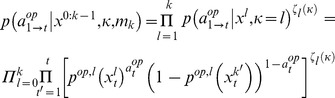
(5)where 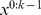 lumps the volatility and temperature of all possible sophistication levels of 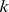-ToM’s opponent, and 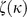 is the indicator vector of the opponent’s ToM level 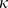 (i.e. 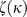 is a 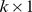 null vector, except 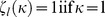). Note that 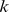-ToM’s generative model 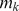 includes the above likelihood function, as well as priors 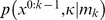 on his opponent’s ToM sophistication level 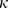 and the observation/evolution parameters 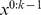 for all levels 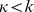. At each trial 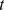, these likelihood and priors induce a free energy bound 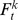 on 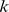-ToM’s (log-) evidence 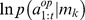 of his opponent’s behaviour:
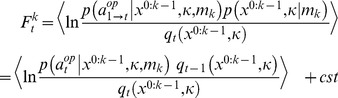
(6)where the expectation is taken under 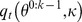, the conditional density on 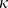 and 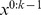 at trial 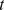, which captures 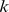-ToM’s posterior belief on her opponent’s properties. The second line of Equation 6 derives from the factorization of the likelihood across time or trials (cf. Equation 4). Variational Bayesian update rules follow from optimizing the free energy with respect to the conditional density 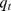 [50]. Without any additional constraint, this yields Bayes rule, i.e.:



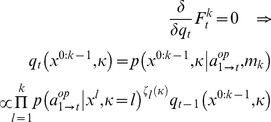
(7)
Equation 7 describes Bayesian (on line) recognition or learning, i.e. how the previous belief 
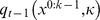
 at trial 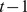 is updated to yield 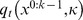, after having observed the opponent’s choice 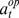 at trial 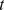. Equation 7 obtains because maximizing the free energy with respect to 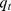 indirectly minimizes the Kullback-Leibler divergence between 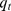 and the posterior density. This means that, without loss of generality, we can rewrite Bayes’ rule in terms of the trial-to-trial evolution of the sufficient statistics 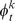 of the time-dependent conditional density 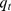 [26]:
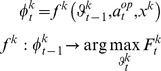
(8)where we have introduced 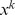, the set of variables that parameterize e.g., 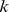 *-*ToM’s prior belief 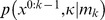 on his opponent (see Appendix 1). In Equation 8, the form of the evolution function 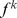 is determined by the Free Energy 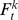, which derives from 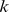 *-*ToM’s generative model 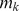. The appeal of this variational formulation is that, under some simplifying assumption about the form of the approximate posterior [51], Bayesian learning becomes analytic. In brief, we have shown how to derive the learning rule of any ToM sophistication level recursively, i.e. from that of the level above. Except for 0-ToM agents, the belief evolution function has the following form (Text S1 for derivation):
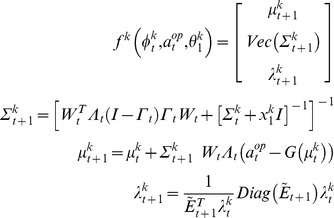
(9)where 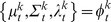 are the sufficient statistics of the time-dependent conditional density 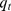, 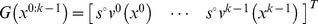 is a 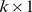 vector composed of the sigmoid observation mappings for each potential ToM sophistication level of 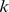-ToM’s opponent (cf. Equation 3), 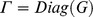, 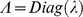, 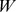 and 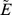 are analytic (matrix and vector) functions of the two moments 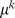 and 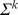. More precisely, 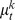 and 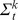 are the first- and second-order moments of the probabilistic belief on 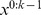, whereas 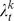 is the first-order moment of the probabilistic belief on 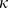 (i.e.: 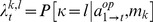). Although Equation 9 is slightly more complex than Equation 2, note that learning is still driven by a simple prediction error term. More precisely, one can see that 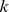-ToM’s prediction error 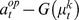 drives the change in her belief sufficient statistics 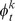. Critically however, this prediction error is weighted by her current belief about her opponent’s sophistication level. Equation 9 is but a compact formulation of how the summary statistics (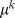, 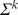 and 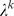) of 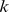-ToM’s posterior distribution 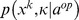 evolve from trial to trial. Both Equations 2 and 9 have been derived using a variational approach to approximate Bayesian inference [51]–[52]. We refer the interested reader to the Text S1.

Equation 3 concludes the exposition of our meta-bayesian model of ToM agents. In brief, we have defined ToM sophistication levels recursively, in terms of their respective (social) learning rule. A critical feature of this meta-Bayesian model is that the complexity of the scenarios that a 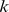-ToM agent uses to learn increases with 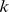. This means that the relative performance of different ToM sophistication levels playing against each other is non-trivial and cannot be evaluated without resorting to computational simulations.

### The Adaptive Fitness of ToM Sophistication Levels

Recall that the adaptive fitness results from the relative behavioural performance of competing phenotypes, which proxies their ability to survive and reproduce [53]. Critically, we view ToM levels as social learning phenotypes that compete with each other (in a Darwinian sense). This differs from standard EGT models, in which phenotypes are defined in terms of their decision policy or strategy (e.g. playing “tit for tat” in the prisoner’s dilemma, [30], [54]. However, this does not invalidate the use of standard EGT replicator dynamics. These describe the evolution of the frequency distribution of competing phenotypes over evolutionary time, given how well they perform when interacting with each other [55]. Let 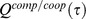 be the 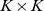 game-dependent expected payoff matrix after 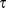 repetitions, where 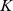 is the maximum ToM sophistication level within the (human) population. The matrix element 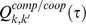 is the expected payoff of a 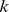-ToM agent playing against a 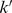-ToM agent. It is obtained by first integrating the system of coupled ToM agents, i.e. iterating forward in time the learning (Equation 2 or 3) and decision (Equation 1) rules up to trial 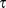, and then measuring the accumulated payoff for each player. The expected payoff is then defined as the Monte-Carlo average of the accumulated payoff over multiple repetitions of the iterated game, where games may yield different outcomes due to the probabilistic nature of the decision policy. On average (across games), the payoff matrix that summarizes the pairwise interaction of individuals is: 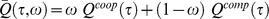, where 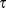 is the number of game repetitions and 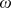 is the probability, for any pair of agents, to engage in a cooperative social interaction. We inserted this average payoff matrix in replicator dynamics to derive the ToM evolutionary stable states. We refer the interested reader to Text S1 for details regarding our implementation of EGT.

## Supporting Information

Text S1 This note provides technical details about the derivation of the learning rule of ToM agents and our application of Evolutionary Game Theory (EGT) to ToM sophistication phenotypes.
(DOCX)
