# Supplementary material for: Theory of Mind: Did Evolution Fool Us?
Source: PLoS One. 2014 Feb 5;9(2):e87619. doi: 10.1371/journal.pone.0087619 (PMC3914827; doi:10.1371/journal.pone.0087619)
Supplement: Text S1 — This note provides technical details about the derivation of the learning rule of ToM agents and our application of Evolutionary Game Theory (EGT) to ToM sophistication phenotypes. (DOCX) [file pone.0087619.s001.docx]

The evolution of theory of mind: did evolution fool us?

SUPPLEMENTARY INFORMATION

*M. Devaine^1^, G. Hollard^2^, J. Daunizeau^1,2^*

*^1^* Brain and Spine Institute, Paris, France

*^2^* Maison des Sciences Economiques, Paris, France

*^3^* Wellcome Trust Centre for Neuroimaging, University College London, United Kingdom

**Key words**: social interaction, competition, cooperation, learning, recursive thinking, evolutionary game theory

Address for correspondence:

Jean Daunizeau

Motivation, Brain and Behaviour Group

Brain and Spine Institute

47, bvd de l’Hopital, 75013, Paris, France.

Tel: +33 1 57 27 43 26

Fax: +33 1 57 27 47 94

Mail: [jean.daunizeau@gmail.com](mailto:jean.daunizeau@gmail.com)

Web: [http://sites.google.com/site/jeandaunizeauswebsite](https://sites.google.com/site/jeandaunizeauswebsite)

This note is organized as follows. In the first section, we expose the derivation of the learning rule of ToM agents (Equations 2 and 9 in the main text). In the second section, we provide details regarding our application of Evolutionary Game Theory (EGT) to ToM sophistication phenotypes.

**Deriving the meta-Bayesian learning rule of ToM observers**

In this section, we will expose the details of the derivation of the variational Bayesian (VB) update rule of ToM observers. Recall that, except for 0-ToM, $k$-ToM's learning rule explicitly derives from the learning rules of ToM observers with levels smaller than $k$ (cf. Equations 1 to 8 in the main text).

- 0-ToM agents.

We will first derive 0-ToM's learning rule, as it is slightly different from any other ToM sophistication level. This will also be helpful to generalize to more sophisticated ToM agents. In the following, we will posit that 0-ToM observers *a priori* believe that the probability of her opponent’s choice may vary smoothly over time, i.e. $p^{op}\equiv p_{t}^{op}$. For numerical reasons, the corresponding prior transition density is defined on log-odds rather than probabilities themselves, i.e.:

$\begin{matrix} p\left(x_{t+1}^{-1}\left|x_{t}^{-1},m_{0}\right.\right)=N\left(x_{t}^{-1},x_{1}^{0}\right) \\ p^{op}\equiv s\left(x_{t}^{-1}\right) \end{matrix}$ (A1)

where $s:x\to1/1+\exp\left(-x\right)$ is the sigmoid mapping, and we have used the notation $x_{t}^{-1}$ to be consistent with the recursive notation of the ToM levels (here, the “level -1” refers to an unsophisticated agent without intentions nor beliefs). In addition, $x_{1}^{0}$ is the prior volatility of 0-ToM’s opponent log-odds $x_{t}^{-1}$. Note that $x_{1}^{0}$ is known to 0-ToM and does not have to be learned. We assume that 0-ToM learns similarly to a Kalman filter (see, e.g., Daunizeau et al., 2009b), by assimilating new observations recursively in time or trials, as follows:

$\begin{matrix} p\left(x_{t+1}^{-1}\left|a_{1\to t}^{op},m_{0}\right.\right)=\int q\left(x_{t}^{-1}\right)p\left(x_{t+1}^{-1}\left|x_{t}^{-1},m_{0}\right.\right)dx_{t}^{-1}\begin{matrix} \\ \end{matrix} \\ q\left(x_{t+1}^{-1}\right)\propto p\left(a_{t+1}^{op}\left|x_{t+1}^{-1},m_{0}\right.\right)p\left(x_{t+1}^{-1}\left|a_{1:t}^{op},m_{0}\right.\right) \end{matrix}$ (A2)

where $q\left(x_{t+1}^{-1}\right)\equiv p\left(x_{t+1}^{-1}\left|a_{1:t+1}^{op},m_{0}\right.\right)$ is the posterior belief about the log-odds $x_{t+1}^{-1}$, conditional upon observed actions up to trial $t+1$. The first line of Equation A2 is 0-ToM's prediction about her opponent’s behavioural tendency. Let us assume that 0-ToM holds a Gaussian probabilistic belief $q\left(x_{t}^{-1}\right)=N\left(\mu_{t}^{0},\Sigma_{t}^{0}\right)$ about the log-odds at trial $t$, where $\mu_{t}^{0}$ and $\Sigma_{t}^{0}$ are the first- and second-order moment of $q$. This implies that her prediction is Gaussian as well, with inflated second-order moment (cf. Equation A1), i.e.: $p\left(x_{t+1}^{-1}\left|a_{1\to t}^{op},m_{0}\right.\right)=N\left(\mu_{t}^{0},\Sigma_{t}^{0}+x_{1}^{0}\right)$. This yields the following expression for the next (log-) posterior density $L\left(x_{t+1}^{-1}\right)\equiv\log q\left(x_{t+1}^{-1}\right)$ (Daunizeau et al. 2010b):

$L\left(x_{t+1}^{-1}\right)=-\frac{1}{2}\frac{1}{\Sigma_{t}^{0}+x_{1}^{0}}\left(x_{t+1}^{-1}-\mu_{t}^{0}\right)^{2}+\log s\left(x_{t+1}^{-1}\right)+\left(a_{t+1}^{op}-1\right)x_{t+1}^{-1}+cst$ (A3)

where the constant is the log-normalization factor. The first iteration of the Laplace approximation (Friston et al., 2007) consists in approximating $L$ by its second-order Taylor expansion around $\mu_{t}^{0}$, and deriving the approximate first- and second-order moments of the corresponding Gaussian density from there on, as follows:

$\begin{matrix} L\left(x_{t+1}^{-1}\right)\approx L\left(\mu_{t}^{0}\right)+L'\left(\mu_{t}^{0}\right)\left(x_{t+1}^{-1}-\mu_{t}^{0}\right)+\frac{1}{2}L''\left(\mu_{t}^{0}\right)\left(x_{t+1}^{-1}-\mu_{t}^{0}\right)^{2} \\ \Rightarrow q\left(x_{t+1}^{-1}\right)\approx N\left(\mu_{t+1}^{0},\Sigma_{t+1}^{0}\right):\left\{\begin{matrix} \mu_{t+1}^{0}=\mu_{t}^{0}-L''\left(\mu_{t}^{0}\right)^{-1}L'\left(\mu_{t}^{0}\right) \\ \Sigma_{t+1}^{0}=-L''\left(\mu_{t}^{0}\right)^{-1} \end{matrix}\right. \end{matrix}$ (A4)

where the derivatives of $L$ are evaluated at $\mu_{t}^{0}$:

$\begin{matrix} L'\left(\mu_{t}^{0}\right)=a_{t+1}^{op}-s\left(\mu_{t}^{0}\right) \\ L''\left(\mu_{t}^{0}\right)=-\frac{1}{\Sigma_{t}^{0}+x_{1}^{0}}-s'\left(\mu_{t}^{0}\right) \\ s'\left(\mu_{t}^{0}\right)=s\left(\mu_{t}^{0}\right)\left(1-s\left(\mu_{t}^{0}\right)\right) \end{matrix}$ (A5)

The limitations of such “early-stopping” variant of the Laplace approximation are discussed in Mathys et al., (2011). Inserting Equation A5 into Equation A4 yields 0-ToM's learning rule:

$\begin{matrix} \mu_{t+1}^{0}=\mu_{t}^{0}+\Sigma_{t+1}^{0}\left(a_{t+1}^{op}-s\left(\mu_{t}^{0}\right)\right) \\ \Sigma_{t+1}^{0}=\left(\frac{1}{\Sigma_{t}^{0}+x_{1}^{0}}+s'\left(\mu_{t}^{0}\right)\right)^{-1} \end{matrix}$ (A6)

where the right-hand term is the explicit form of the evolution function of 0-ToM's belief sufficient statistics .

- Inferring belief and preferences: $k$-ToM agents ($k\geq1$)

Similarly to Equation A1, we posit that $k$-ToM observers *a priori* believe that the parameters of any level-$k'$ player ($k'<k$) may vary smoothly over time, yielding the following prior transition density:

$\begin{matrix} p\left(x_{t+1}^{0:k-1}\left|x_{t}^{0:k-1},m_{k}\right.\right)=N\left(x_{t}^{0:k-1},x_{1}^{k}R\right) \\ x_{t}^{0:k-1}=\left[\begin{matrix} x_{t}^{0} \\ \vdots\\ x_{t}^{k-1} \end{matrix}\right] \end{matrix}$ (A7)

where $x_{t}^{0:k-1}$ is the set of evolution and observation parameters of ToM players of levels $k'<k$, and $R$ is a fixed covariance matrix of random innovations (see below), which is scaled by the prior volatility $x_{1}^{k}$ of the stochastic dynamics of $x^{0:k-1}$. In contradistinction, the level of her opponent is assumed to be static over time. A natural prior for the opponent’s level is thus the following multinomial distribution:

(A8)

where $\zeta\left(\kappa\right)$ is the indicator $k\times1$ vector of the opponent's level $\kappa$ (i.e. $\zeta_{l}\left(\kappa\right)=1\text{ iif }\kappa=l$), and $\lambda_{0}^{k,l}=P\left(\kappa=l\left|m_{k}\right.\right)$ obey a normalization constraint, i.e.: $1=\sum_{l=0}^{k-1} \lambda_{0}^{k,l}$. Equations A7 and A8 can be inserted into Equation 6 of the main text to form the free energy bound $F_{t}^{k}$ on the (log-) model evidence of the $k$-ToM observer. This free energy is optimized under a so-called mean-field assumption, yielding:

$q\left(x^{0:k-1},\kappa\right)\approx q\left(x^{0:k-1}\right)q\left(\kappa\right)\mathop{\Rightarrow}\limits^{\delta F/\delta q=0} \left\{\begin{matrix} q\left(x^{0:k-1}\right)\propto\exp\left\langle L\left(x^{0:k-1},\kappa\right)\right\rangle_{q\left(\kappa\right)} \\ q\left(\kappa\right)\propto\exp\left\langle L\left(x^{0:k-1},\kappa\right)\right\rangle_{q\left(x^{0:k-1}\right)} \end{matrix}\right.$ (A9)

where the log-joint $L\left(x^{0:k-1},\kappa\right)=\log p\left(a_{1:t}^{op},x^{0:k-1},\kappa\left|m_{k}\right.\right)$ is given by the likelihood (Equation 5 in the main text) and the priors (Equations A7 and A8).

First, let us consider the approximate conditional density $q\left(x^{0:k-1}\right)$ on evolution and observation parameters of ToM agents of levels $k'<k$. As for 0-ToM, assume that $k$-ToM holds a Gaussian belief $q\left(x_{t}^{0:k-1}\right)=N\left(\mu_{t}^{k},\Sigma_{t}^{k}\right)$ about the set of evolution and observation parameters of ToM players of levels $k'<k$. This implies that her prediction is Gaussian as well, with inflated second-order moment, i.e.: $p\left(x_{t+1}^{0:k-1}\left|a_{1\to t}^{op},m_{0}\right.\right)=N\left(\mu_{t}^{k},\Sigma_{t}^{k}+x_{1}^{k}R\right)$. This yields the following expression for the expected (log-) joint $\tilde{L}\left(x^{0:k-1}\right)\equiv\left\langle L\left(x^{0:k-1},\kappa\right)\right\rangle_{q\left(\kappa\right)}$:

$\begin{matrix} \tilde{L}\left(x_{t+1}^{0:k-1}\right)=\lambda_{t}^{T}\left(a_{t+1}^{op}\log G\left(x_{t+1}^{0:k-1}\right)+\left(1-a_{t+1}^{op}\right)\log\left(1-G\left(x_{t+1}^{0:k-1}\right)\right)\right) \\ +\frac{1}{2}\left(x_{t+1}^{0:k-1}-\mu_{t}^{k}\right)^{T}\left[\Sigma_{t}^{k}+x_{1}^{k}R\right]^{-1}\left(x_{t+1}^{0:k-1}-\mu_{t}^{k}\right)+cst \\ G\left(x_{t+1}^{0:k-1}\right)\equiv\left[\begin{matrix} s\circ v^{0}\left(x_{t+1}^{0}\right) \\ \vdots\\ s\circ v^{k-1}\left(x_{t+1}^{k-1}\right) \end{matrix}\right] \\ \lambda_{t}^{k}\equiv\left\langle\zeta\left(\kappa\right)\right\rangle=\left[\begin{matrix} q_{t}\left(\kappa=0\right) \\ \vdots\\ q_{t}\left(\kappa=k-1\right) \end{matrix}\right] \end{matrix}$ (A10)

where $\lambda_{t}^{k}$ is a $k\times1$ vector containing the previous posterior expectation on the opponent’s ToM level (i.e.:$\lambda_{t}^{k,l}=P\left(\kappa=l\left|a_{1\to t}^{op},m_{k}\right.\right)$), and $G\left(x_{t+1}^{0:k-1}\right)$ is a $k\times1$ vector made of the prediction of the opponent’s choice under each and every subordinate ToM sophistication level. Note that $G$ is an implicit function of the evolution parameters through the incentive strength $\Delta V_{t+1}^{l}\equiv\Delta V\left(x_{1,t+1}^{l}\right)$. In fact, $G_{l}\left(x_{t+1}^{0:k-1}\right)$ is the composition of the sigmoid mapping (cf. Equation 1) with a nonlinear function $v^{l}$ of $l$-ToM's evolution and observation parameters, i.e.:

$\begin{matrix} G_{l}\left(x_{t+1}^{0:k-1}\right)=s\circ v^{l}\left(x_{t+1}^{l}\right) \\ v^{l}\left(x_{t+1}^{l}\right)=\frac{\Delta V\left(x_{1,t+1}^{l}\right)}{x_{2,t+1}^{l}} \\ \Delta V\left(x_{t+1}^{l}\right)=p^{op}\left(x_{t+1}^{l}\right) \Delta U\left(a^{op}=1\right)+\left(1-p^{op}\left(x_{t+1}^{l}\right)\right) \Delta U\left(a^{op}=0\right) \\ \Delta U\left(a^{op}=j\right)=U\left(a^{self}=1,a^{op}=j\right)-U\left(a^{self}=0,a^{op}=j\right) \end{matrix}$ (A11)

Equation A11 can now be inserted into Equation A9 to derive the first and second derivatives of the expected (log-) joint $\tilde{L}\left(x^{0:k-1}\right)$:

$\begin{matrix} \tilde{L}'\left(\mu_{t}^{k}\right)=W_{t}\Lambda_{t}\left(a_{t+1}^{op}-G\left(\mu_{t}^{k}\right)\right) \\ \tilde{L}''\left(\mu_{t}^{k}\right)=-W_{t}^{T}\Lambda_{t}\left(I-\Gamma_{t}\right)\Gamma_{t}W_{t}-\left[\Sigma_{t}^{k}+x_{1}^{k}R\right]^{-1} \end{matrix}$ (A12)

where $\Lambda=Diag\left(\lambda\right)$, $\Gamma=Diag\left(G\right)$, and $W$ is the gradient matrix of the functions $v$:

$\begin{matrix} W_{t}=\left[\begin{matrix} \frac{\partial v^{0}}{\partial x_{t+1}^{0:k-1}} & \cdots& \frac{\partial v^{k-1}}{\partial x_{t+1}^{0:k-1}} \end{matrix}\right] \\ \frac{\partial v^{l}}{\partial x_{t+1}^{0:k-1}}=\frac{1}{x_{2,t+1}^{l}}I_{l}\otimes\left[\begin{matrix} \frac{\partial\Delta V_{t+1}^{l}}{\partial x_{1,t+1}^{k'}} \\ -\frac{1}{2}\frac{\Delta V_{t+1}^{l}}{x_{2,t+1}^{l}} \end{matrix}\right] \end{matrix}$ (A13)

where denotes the Kronecker product, and $I_{l}$ is the $l$^th^ column of the identity matrix. Similarly to 0-ToM agents, an “early-stopping” Laplace approximation yields the following update rule for the first- and second-order moments of $q\left(x_{t+1}^{0:k-1}\right)$:

$\begin{matrix} \Sigma_{t+1}^{k}=\left[W_{t}^{T}\Lambda_{t}\left(I-\Gamma_{t}\right)\Gamma_{t}W_{t}+\left[\Sigma_{t}^{k}+x_{1}^{k}R\right]^{-1}\right]^{-1} \\ \mu_{t+1}^{k}=\mu_{t}^{k}+\Sigma_{t+1}^{k} W_{t}\Lambda_{t}\left(a_{t+1}^{op}-G\left(\mu_{t}^{k}\right)\right) \end{matrix}$ (A14)

where all gradients are evaluated at $\mu_{t}^{k}$. Equation A14 is similar in form to Equation A5, except in terms of the non-trivial impact of the probabilistic belief on the opponent’s ToM level. Note that appropriately nulling elements in the transition covariance matrix $R$ allows one to model agents that assume the temporal invariance of certain hidden parameters (e.g., action emission temperature $x_{2}$). Equation 9 in the main text corresponds to $R=I$.

Now we turn to the conditional density on the opponent’s ToM level, i.e. $q\left(\kappa\right)$. First, recall that $k$-ToM holds a multinomial belief, whose sufficient statistics at trial $t$ is $\lambda_{t}^{k}$. Deriving the update rule for $q\left(\kappa\right)$ relies on deriving the expected (log-) joint $\tilde{L}\left(\kappa\right)\equiv\left\langle L\left(x^{0:k-1},\kappa\right)\right\rangle_{q\left(x^{0:k-1}\right)}$, which depends on the first- and second-order moments of $q\left(x^{0:k-1}\right)$:

$\tilde{L}\left(\kappa\right)=\zeta\left(\kappa\right)^{T}\left(a_{t+1}^{op}\left\langle\log G\left(x_{t+1}^{0:k-1}\right)\right\rangle+\left(1-a_{t+1}^{op}\right)\left\langle\log\left(1-G\left(x_{t+1}^{0:k-1}\right)\right)\right\rangle\right)+\zeta\left(\kappa\right)^{T}\log\lambda_{t}^{k}+cst$ (A15)

This requires an approximation to $\left\langle\log G\left(x_{t+1}^{0:k-1}\right)\right\rangle$, where the expectation is taken under $q\left(x_{t+1}^{0:k-1}\right)$. Using Equation A11, one can show that the expected log-sigmoidal mapping can be well approximated as follows:

(A16)

where $\tilde{v}^{l}$ is the mapping of sufficient statistics $\mu_{t+1}^{l}$ and $\Sigma_{t+1}^{l}$ that furnishes an accurate approximation of the expected log sigmoid, and the values of constants $c_{1,2,3}$ have been determined numerically ($c_{1}=-0.41$,$c_{2}=0.72$,$c_{3}=0.11$). Equation A16 can be inserted into Equation A15 to yield:

$\begin{matrix} \tilde{L}\left(\kappa\right)=\sum_{l=0}^{k-1} \zeta\left(\kappa\right)\log\left(\lambda_{t,l}^{k}+\tilde{E}_{t+1}^{l}\right)+cst \\ \tilde{E}_{t+1}^{l}=s\circ\tilde{v}^{l}\left(\mu_{t+1}^{l},\Sigma_{t+1}^{l}\right)\exp\left[-\left(1-a_{t+1}^{op}\right)v^{l}\left(\mu_{t+1}^{l}\right)\right] \end{matrix}$ (A17)

Equation A17 has a (log-) multinomial functional form, where $\tilde{E}_{t+1}$ acts as a correction term on the previous sufficient statistics $\lambda_{t}^{k}$. In fact, this can be used to rewrite the learning rule on the opponent’s level as follows:

$\lambda_{t+1}^{k}=\frac{1}{\tilde{E}_{t+1}^{T}\lambda_{t}^{k}}Diag\left(\tilde{E}_{t+1}\right)\lambda_{t}^{k}$ (A18)

where we have accounted for the normalization constraint of the multinomial distribution.

This concludes the theoretical recursive construct of $k$-ToM agents. In brief, 0-ToM (Bayesian) agents adapt their behaviour based upon their expectation about their opponent’s choices, which they learn over the course of the game. A 1-ToM (meta-Bayesian) agent takes the intentional stance on her 0-ToM opponent, i.e. learns the hidden priors that shape 0-ToM agents’ behaviour. More generally, a $k$-ToM agent assumes she faces an opponent with unknown ToM sophistication level $\kappa<k$, which has to be learned (in addition to the ensuing prior beliefs). The recursive construction of ToM sophistication levels can be seen as an analytic mapping from a $k$-ToM learning rule to a $\left[k+1\right]$-ToM learning rule. Importantly, the construction of this mapping is neither game-dependent nor level-dependent. The former point is important, because it means that the above model captures agents that can be thought of as experts in reciprocal social interaction, irrespective of what is at stake. The latter point means that, at his stage, there is no theoretical limit to the ToM sophistication level.

Last but not least, note that increasing ToM sophistication induces a non-trivial statistical cost, in terms of the expected error when predicting the opponent's next move. This is because the number of unknown variables in the generative model of a $k$-ToM agent grows super-linearly with its sophistication level $k$. Since model variables are not perfectly identifiable, this basically increases the mean expected estimation error[[1]](#footnote-1). In turn, this compromises the precision of $k$-ToM's prediction $p^{op}$ about her opponent's next move. This cost to sophistication will turn out to be critical when evaluating the adaptive fitness of ToM agents.

**Adaptive fitness of ToM sophistication levels**

If there is no theoretical limit to the ToM sophistication level, why aren’t we all capable of infinite (or very large) ToM recursion in the context of social interaction? We assume that the effective bound on ToM sophistication is the consequence of evolutionary pressure that acted on ToM phenotypes. In this section, we adapt standard EGT replicator dynamics to model the Darwinian competition of ToM sophistication levels. In brief, replicator dynamics describe the evolution of frequencies of phenotypes within the population over evolutionary time, as a function of their respective performance in the context of ecological games that capture inter-individual cooperation and/or competition. The key point here is that the evolutionary success of a strategy is not just determined by how good is the strategy (compared with another strategy), it is also a function of how frequent are all the strategies within a competitive population. Of particular importance is how good a strategy plays against itself, because a successful strategy will eventually dominate and competing individuals will face identical strategies to their own.

Let $s_{k}\left(t\right)$ be the proportion of $k$-ToM agents within the population at evolutionary time $t$, with $0\leq k\leq K$, where $K$ is the maximum ToM level within the population. At each time (or generation), individuals meet in pairwise contests with others, where each interaction is a repeated game. Note that we will be concerned with more than one type of game (see below). Let $Q^{(i)}\left(\tau\right)$ and $\omega_{i}$ be, respectively, the $K\times K$ expected payoff matrix of the $i$^th^ type of game after $\tau$ repetitions, and the associated probability for any pair of agents to play the $i$^th^ type of game. The matrix element $Q_{k,k'}^{(i)}\left(\tau\right)$ is the expected payoff of a $k$-ToM agent playing the $i$^th^ type of game against a $k'$-ToM agent. It is obtained by first integrating the system of coupled ToM agents, i.e. iterating forward in time the evolution (Equation 1) and observation (Equation 3) processes up to trial $\tau$, and then measuring the accumulated payoff for each player. The expected payoff is then defined as the Monte-Carlo average of the accumulated payoff over multiple repetitions of the iterated game, where games may yield different outcomes due to the probabilistic nature of the action emission law[[2]](#footnote-2). Thus, on average (across games), the expected payoff matrix $\bar{Q}\equiv\bar{Q}\left(\tau,\omega\right)$ that summarizes the pairwise interaction of individuals after $\tau$ game repetitions is:

$\bar{Q}\left(\tau,\omega\right)=\sum_{i} \omega_{i} Q^{(i)}\left(\tau\right)$ (A19)

Standard EGT replicator dynamics derives from assuming that (Morgan and Steiglitz 2003): (i) the absolute fitness of an agent is the average payoff it receives, (ii) the probability that an agent of type $k$ interacts with any other agent in a small time interval $dt$ is proportional to its proportion $s_{k}\left(t\right)dt$, within the population, and (iii) the change $ds_{k}$ in the proportion of agents of type $k$ (during $dt$) is proportional to their relative fitness (i.e. their absolute fitness minus the average fitness of the entire population). This yields:

$\frac{ds}{dt}=Diag\left(s\right)\left(\bar{Q}s-s^{T}\bar{Q}s\right)$ (A20)

This ordinary differential equation, referred to by Hofbauer and Sigmund (1998) as the “basic tenet of Darwinism”, describes how the proportion $s\left(t\right)$ of (here, ToM) phenotypes within a population evolves under evolutionary pressure. Fixed points $s*$ of this equation describe *evolutionary stable states*, i.e. a repartition of phenotypes that is restored by selection after a disturbance, provided the disturbance is not too large (Maynard-Smith, 1982). One can see how evolutionary dynamics can change as a function of the expected payoff matrix $\bar{Q}\left(\tau,\omega\right)$. Thus, the adaptive fitness of ToM sophistication levels may be a non-trivial function of games duration $\tau$ and frequency $\omega$. In fact, we will show that this dependency is critical, in that it determines the ToM sophistication level that is selected by evolution.

1. More technically, one can show that the so-called bayesian Cramer-Rao bound (the lower bound on the mean squared estimation error) increases as ToM sophistication increases. [↑](#footnote-ref-1)
2. Actions at each trial are randomly sampled according to the probabilistic emission law given in Equation 1 of the main text. [↑](#footnote-ref-2)
